# Feasibility of non-invasive recording of somatosensory evoked potential in pigs

**DOI:** 10.1186/s42826-022-00118-3

**Published:** 2022-03-24

**Authors:** Guillaume L. Hoareau, Angela Peters, David Hilgart, Marta Iversen, Gregory Clark, Matthew Zabriskie, Viola Rieke, Candace Floyd, Lubdha Shah

**Affiliations:** 1 Emergency Medicine, Department of Surgery, University of Utah, Salt Lake City, UT USA; 2 Department of Neurology, University of Utah, Salt Lake City, UT USA; 3 Department of Biomedical Engineering, University of Utah, Salt Lake City, UT USA; 4 Department of Radiology and Imaging Sciences, University of Utah, Salt Lake City, UT USA; 5 Department of Physical Medicine and Rehabilitation, University of Utah, Salt Lake City, UT USA

**Keywords:** Large animal model, Butorphanol, Dexmedetomidine, Electroencephalogram, Electroencephalography, Midazolam, Translational research

## Abstract

**Background:**

Non-invasive measurement of somatosensory-evoked potentials (SEP) in a large animal model is important to translational cognitive research. We sought to develop a methodology for neurophysiological recording via a transcranial electroencephalography (EEG) cap under an effective sedative regimen with dexmedetomidine, midazolam, and butorphanol that will produce sedation instead of anesthesia while not compromising data quality.

**Results:**

Pigs received intramuscular dexmedetomidine, midazolam, and butorphanol for SEP assessment with peroneal nerve stimulation. Semi-quantitative sedation assessment was performed after the animal was sufficiently sedated and 30 min later, during the transcranial SEP recording. SEP data were analyzed with commercial software. Binary qualitative analysis of the recording was categorized by an experienced neurophysiologist. All four animals had adequate surface SEP recordings. Animals received 43 [21–47] mcg/kg of dexmedetomidine, 0.3 [0.2–0.3] mg/kg of midazolam, and 0.3 [0.3–0.3] mg/kg of butorphanol IM. All treatments resulted in moderate to deep sedation (Baseline median sedation score 11.5 [11–12]; median score at 30 min: 11.5 [10.5–12]). Heart rate (median [range]) (55 [49–71] beats per minute), respiratory rate (24 [21–30] breaths per minute), and hemoglobin oxygen saturation (99 [98–100]%) and body temperature (37.7 [37.4–37.9] °C) remained within clinically acceptable ranges. There were no undesirable recovery incidents.

**Conclusions:**

In this pilot study, we demonstrate the feasibility of SEP recording via a transcranial EEG cap under an effective sedative regimen in pigs. Our approach will expand the use of a large animal model in neurotranslational research.

**Supplementary Information:**

The online version contains supplementary material available at 10.1186/s42826-022-00118-3.

## Background

Animal models of neurological disorders are an invaluable translational tool, providing information on novel techniques and treatments that may not be as readily obtained from patients. Discovery efforts that utilize animal models allow for control of various variables and reproducibility of results. Electrophysiological neuromonitoring methods, such as electroencephalography (EEG) and somatosensory-evoked potentials (SEP), provide crucial insight into the functional integrity of neural structures. Although very informative, invasive methods of measuring cortical activity with stimulation, such as skull screws and electrocorticography [1–3] are challenging to implement clinically routinely and have less translational potential to demonstrate novel findings in animals. Non-invasive measures, such as EEG, are therefore advantageous to accelerate clinical discovery.

Brain electrophysiological research using EEG and SEP to study cognitive processing in animals needs to be non- or minimally-invasive, painless, and reproducible to accelerate translation to humans. In animals, especially pigs, there is an increased impedance level due to the thicker calvarium and skin compared to humans. Physiologic factors, such as temperature, blood pressure, hematocrit, acid–base balance, and oxygen and carbon dioxide tensions, which are altered with anesthesia, influence SEP data acquisition. Anesthetic drugs and sedatives are the most common pharmacologic causes of nonspecific SEP changes [4] and can affect cognitive processing of the stimulus [5].

Anesthetic and sedative agents are frequently used during electrophysiological studies. They have a dose-dependent adverse effect on the ability to record SEP responses. General anesthesia has an inhibitory effect on neurotransmission and, therefore, on the SEP. Because the anesthetics’ effects are greater on synaptic transmission than on axonal conduction [6], responses recorded from polysynaptic pathways (e.g., cortical recordings) are affected by anesthesia to a much greater extent than those recorded from oligosynaptic pathways (e.g., spinal cord and subcortical recordings) [7]. All volatile anesthetics produce a dose-dependent increase in SEP latency, an increase in central conduction time, a decrease in amplitude and central conduction time [7–13]. Commonly used intravenous anesthetics alter experimental results, although they generally affect SEPs less than inhaled anesthetics. Barbiturates affect synaptic transmission more than axonal conduction and produce a dose-dependent increase in latency and decrease in early cortical SEP amplitude. Propofol’s effect on SEPs is similar to that of the barbiturates; it is also characterized by rapid recovery for timely postprocedural neurologic assessment [4]. When used as a sedative hypnotic in combination with opioids, propofol reduces SEP amplitude less than nitrous oxide or midazolam. Benzodiazepines have only mild-to-moderate depressant effects on SEPs. Benzodiazepines affect sensory pathways differentially. Decreasing amplitude of the evoked electromyelogram response (a spinal cord response to somatosensory stimulation) indicates a peripheral action. Conversely, sedative doses of benzodiazepines leave the early cortical waveforms unaffected [14]. Most authors report clinically unimportant changes in SEP latency and amplitude after administering opioids, whether given in analgesic or anesthetic doses [4]. Ketamine and etomidate increase cortical SEP amplitude without affecting cortical latency [15] or subcortical waveforms [16]. Dexmedetomidine, an α2-receptor agonist, is used widely to produce sedation and analgesia with a dose-dependent sedative and analgesic effects [17]. It has minimal effects on SEP recordings in small animals [18]. However, no published reproducible sedation protocol enables reliable non-invasive, transcranial brain electrophysiological monitoring in large animals.

This pilot study aimed to develop a safe and reproducible methodology for non-invasive neurophysiological recording using a sedation regimen that allows reliable recording of transcranial SEPs in a large animal model.

## Results

### Assessment of SEP quality

A qualitative assessment of the SEP recordings is described in Table 1. All four animals had adequate surface SEP recordings.

**Table 1:** Quality assessment of somatosensory evoked potentials (SEPs)

Pig	Sedation score	SEP assessment
1	12	Definitive
2	12	Definitive
3	10.5	Definitive
4	11	Definitive

SEPs were categorized as inadequate versus definitive. A definitive SEP was defined as excellent waveform quality and signal-to-noise ratio. The sedation score provided is the average of the sedation scores at baseline and 30 min later

### Sedation quality

The median sedation score at time zero (T0) was 11.5 [11, 12], and at 30 min post-induction (T30) was 11.5 [11, 12]. The animals’ physiologic parameters and doses of medication administered are detailed in Table 2. There were no undesirable recovery incidents.

**Table 2:** Animals and sedation characteristics

*Sex*	
Male (N)	0
Female (N)	4
Bodyweight (kg)	27.1 [24.2–36.8]
Time to recumbency (min)	8.4 [3.0–10.3]
Time to standing (min)	71.5 [50.0–105.0]
Mean heart rate (beats/min)	55 [49–71]
Mean respiratory rate (respirations/min)	24 [21–30]
Mean pulse oximetry (%)	99 [98–100]
Mean core temperature (C)	37.7 [37.4–37.9]
Dexmedetomidine (µcg/kg)	43 [21–47]
Midazolam (mg/kg)	0.3 [0.2–0.3]
Butorphanol (mg/kg)	0.3 [0.3–0.3]

Values are presented as median [range]

## Discussion

In this study, we report a reproducible methodology to obtain high-quality transcranial SEP recordings in a large animal model using an effective sedation regimen that provided adequate and safe immobilization.

The pig model is increasingly used in neuroscience because of brain similarities with humans [19, 20]. The major benefit for neuroscience research is the size of the pig brain, which is large enough to allow SEP recordings, neurosurgery, and conventional imaging in live animals. The pig has cerebral structures common to other mammalian species. With relatively well-defined cerebral circumvolutions, its brain appears to be comparable to humans in anatomy, histology, and vascularization [19]. The pig has proven to be a superior experimental animal for SEP recordings, which requires a relatively large brain [19–21]. In contrast to some primates, both auditory and somatosensory cortical regions are located mainly in the gyral surfaces, with little sensory activity in the infolded sulcal regions [22]. Furthermore, the use of pig models is less cost-prohibitive, less dangerous, and poses less of an ethical dilemma than primate models.

### Assessment of SEP quality

SEPs are considered recordable when reproducible waveforms are reported. The SEP waveform consists of a series of peaks and valleys presented as a graph of voltage over time and described in terms of amplitude, latency, and morphology. The amplitude is commonly measured as the waves’ peak-to-peak voltage difference. Latency is the time from stimulus to the peak of the response. The low amplitude cortical sensory evoked response (1–2 microV) has to be extracted from concurrent spontaneous EEG activity (50–100 microV) by repetitive stimulation and computer-signal averaging techniques [23].

Although it has also been possible to obtain SEP from scalp recordings in awake, non-sedated pigs [24], such neurophysiological recording is routinely performed under anesthesia. SEP recording has been informative with isoflurane when performed with skull screw electrodes [25] or electrocorticography [26]. Maier et al. showed adequate SEP monitoring with the pig under general anesthesia with propofol [27]. However, general anesthesia has an inhibitory effect on synaptic neurotransmission and, therefore, on the SEP. Polysynaptic pathways (e.g., cortical recordings) are affected by anesthesia to a much greater extent than those recorded from oligosynaptic pathways [4]. General anesthesia also imparts the risks associated with intubation, such as aspiration pneumonia, difficult or prolonged recovery, atelectasis, etc. This is particularly problematic when repeated SEP recordings sessions are required.

### Sedation quality

We show that the sedation regimen used in this study (dexmedetomidine, midazolam, and butorphanol) provides adequate sedation to obtain excellent SEP recordings from a transcranial EEG cap. Dexmedetomidine mainly inhibits the release of norepinephrine by acting on the α-adrenergic receptor of the brainstem nucleus, producing good sedative effects. We chose dexmedetomidine over other alpha 2-agonists for two reasons: 1- ease of procurement from our vendor, and 2- extensive experience with this drug in both laboratory and clinical settings. Butorphanol has a longer duration of action than fentanyl, and it also has a certain sedative effect. The combination of the two is more conducive to managing postanesthetic agitation and pain. Combining an alpha_2_-adrenergic receptor agonist with an opioid increases the depth and quality of sedation compared with an alpha_2_-adrenergic receptor agonist alone. Midazolam acts as a pre-anesthetic sedative in pigs and allows lower doses of butorphanol. Previous studies have used sedation scores comparing different sedatives [28–30]. We believe further studies are needed to compare our sedation protocol to others. Furthermore, our pigs are ordered in batches for various experiments and animals used for this study happened to be castrated males. Future larger scale studies could include both male and female pigs.

Although an absolute sedation score value has not been described as differentiating between the various degrees of sedation, the median sedation score of 11.5 at T0 and T30 in our study was adequate to maintain animal immobility in the sling and ensure physiological stability. Avoiding a major surgical procedure facilitates serial evaluation of a given subject since there is no morbid intervention such as craniectomy. This ability to assess brain activity in the pig is invaluable for future neurotranslational research.

## Conclusions

In this report, we have demonstrated a novel reproducible method to obtain transcranial SEP recordings in a large animal model using an effective and safe sedation regimen. The modified sedation score scale was adapted from previously published applications in dogs and tracked parameters that are relevant across species. Our methodology will be applied to future investigations for neurophysiological recordings in large animal models. In addition, it will expand the use of pigs in neurotranslational research and accelerate the testing of novel interventions.

## Methods

Four healthy adults castrated male Yorkshire-cross swine (*Sus scrofa*, Premier BioSource, Ramona, CA) were acclimated for a minimum of 7 days and fasted for 12 h before the study. Animals are housed in compatible groups on soft bedding with a 12-h light cycle. They are fed a commercial diet (Teklad swine diet, Envigo, Indianapolis, IN).

### Sedation protocol

All animals were sedated with an intramuscular injection of dexmedetomidine (20–40 ug/kg), midazolam (0.3 mg/kg), and butorphanol (0.3 mg/kg) of estimated body weight. Bodyweight was measured after sedation. Physiologic data, including heart rate, respiratory rate, temperature, and pulse oximetry, were recorded throughout the experiment. Sedation score metrics measured included (modified from Gurney et al. [28]) (Table 3): time from injection to the animal laying down, time from injection to the animal standing, subjective recovery (smooth–acceptable–unacceptable), palpebral reflex, eye position, jaw and tongue relaxation, response to noise, and general appearance/attitude. The sedation score was measured at T0 (first assessment) after induction and at T30 (30 min after T0).

**Table 3:** Modified sedation scoring (modified from Gurney et al. [28])

Sedation scale	Score
*Spontaneous posture*	
Standing	0
Weak but standing	1
Lying but able to rise	2
Lying but difficulty rising	3
Unable to rise	4
*Palpebral reflex*	
Brisk	0
Slow but with full corneal sweep	1
Slow but only partial corneal sweep	2
Absent	3
*Eye position*	
Central	0
Rotated but not obscured by third eyelid	1
Rotated and obscured by third eyelid	2
*Jaw and tongue relaxation*	
Normal jaw tone, strong gag reflex	0
Reduced tone, moderate gag reflex	1
Much reduced tone, slight gag reflex	2
Loss of tone, no gag reflex	3
*Response to noise*	
Normal startle reaction	0
Reduced startle reaction	1
Minimal startle reaction	2
Absent reaction	3
*General appearance/attitude*	
Excitable	0
Awake and normal	1
Tranquil	2
Stuporous	3

**Fig. 1:**
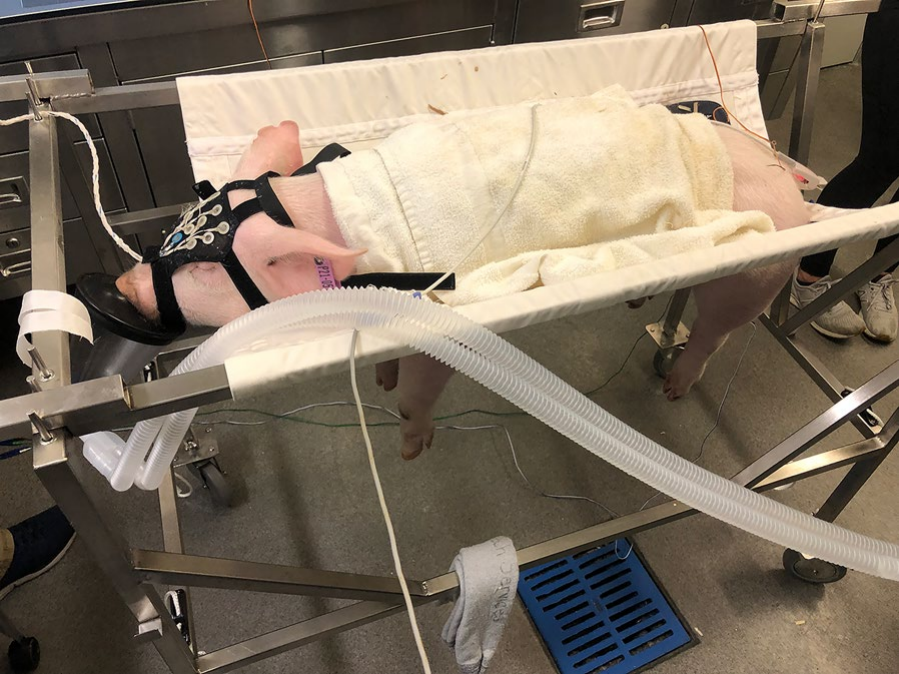
Animal positioning. The animal was placed in a hammock on wheels, the four legs freely hanging through holes, and the head and body resting on the fabric support. The electroencephalography cap on the head is used to record somatosensory-evoked potentials

An ear vein catheter was placed in the event of a complication. The animal was then placed in a hammock on wheels, the four legs freely hanging through holes and the head and body resting on the fabric support (Fig. 1).

### Somatosensory-evoked potential recording

**Fig. 2:**
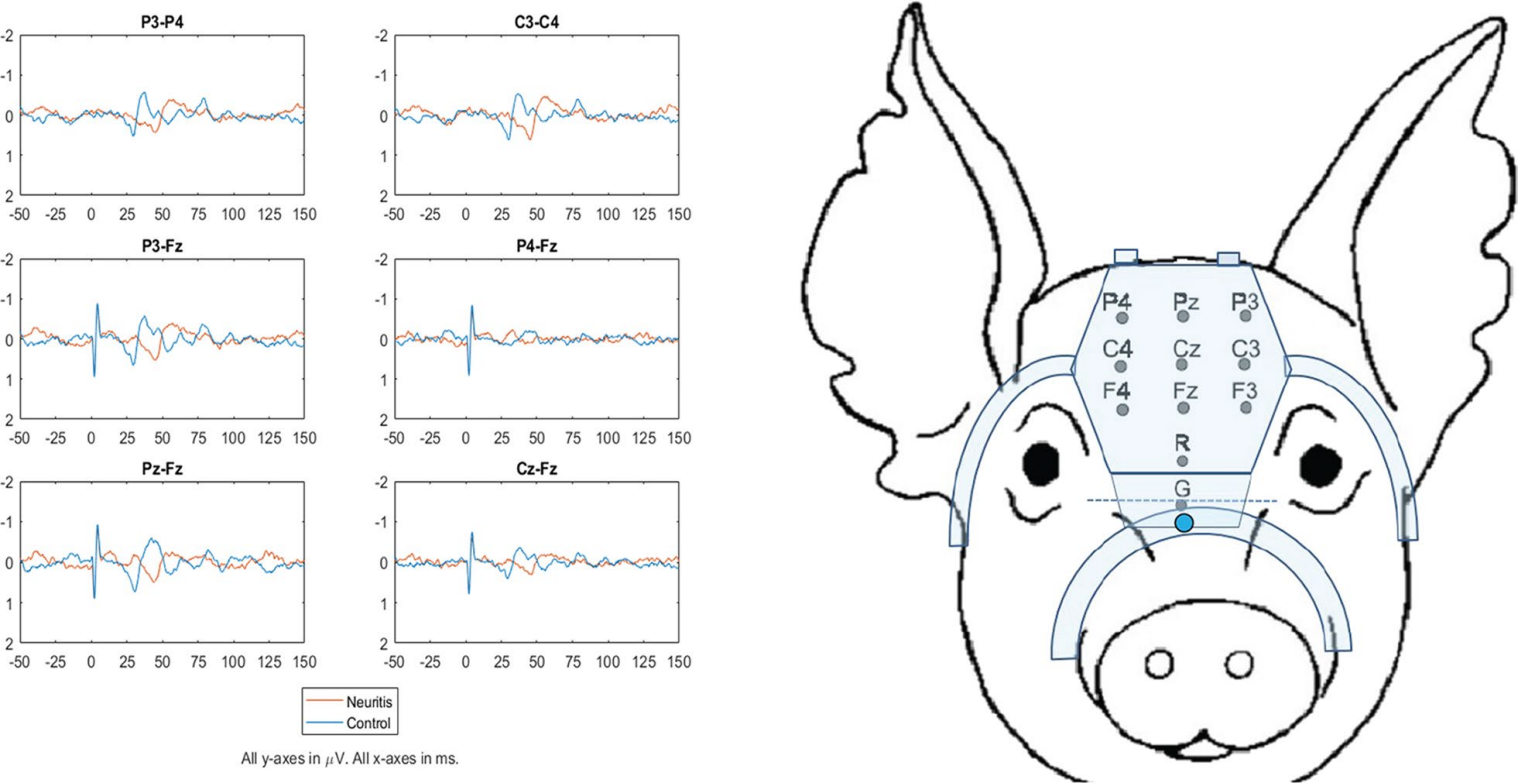
Representative somatosensory evoked potential recording (SEP). Representative SEP recordings 6 weeks after peroneal neuritis induction. Six differential pairs of electrode channels were interrogated and plotted. The stimulus was delivered at 0 ms, where an artifact is seen. Neuritis: Stimulation on the side of the injured peroneal nerve. Control: Stimulation on the contralateral nerve. Stimulation on the neuritis side resulted in similar SEP waveforms but longer latency

Each animal underwent SEP recordings under the aforementioned sedation protocol (Fig. 2). Stimulating pairs of 19 mm subdermal needle electrodes (Rhythmlink International LLC Columbia, South Carolina) were placed into the subcutaneous soft tissues along the course of each lateral peroneal nerve. Transcranial SEP recordings with a custom pig EEG cap (Brain Vision LLC Morrisville, North Carolina) were obtained during alternating lateral peroneal nerve stimulations and averaged together over 800–1200 trials. (Fig. 2) Stimuli were delivered using two Grass Instruments SD9 Stimulators (Astro-Med) set to 200 µs square wave pulses at just under 2.5 Hz, with an approximately 200 ms offset between legs. Stimulation voltage was adjusted to achieve a visibly supramaximal motor response. EEG signals were amplified using a shielded, battery-powered amplifier (Brain Vision LLC Morrisville, North Carolina), recorded with BrainVision Recorder software, and analyzed with BrainVision Analyzer software as well as MATLAB. Each channel’s impedance was measured prior to recording, with additional conductive gel added to any channel with an impedance over 5 kΩ. For SEP recording, we interrogated bipolar pairs of electrodes referring to positions analogous to the human 10/20 system. For instance, we examined the Pz/Fz combination referring to the Pz/Fz in the human 10/20 system, which corresponds to the central sulcus [25]. The overall organization of the primary somatosensory cortex is similar in pigs to that of other mammals [31, 32]. The parameters assessed were amplitude and latency of P30 waves of SEP as the primary outcomes, with “P” describing positive potentials (downward wave) according to international nomenclature. P30 has been previously described as the average latency of the SEP potential of tibial nerve stimulation in porcine [27]. The SEP recordings were reviewed by a neurologist with expertise in neurophysiological assessments and rated as inadequate or definitive, based on the signal-to-noise ratio and the quality of the waveform (Additional file 1).

### Statistical methods

Summary statistics are presented as median [range] (Stata 14.2, StataCorp, TX).

## Supplementary Information

**Additional file 1**. Dataset supporting the conclusions of this article.

## Data Availability

The dataset(s) supporting the conclusions of this article is (are) included within the article (and its additional file 1.

## Abbreviations

EEG: Electroencephalography
SEP: Somatosensory-evoked potential

## References

[1] Ledbetter NM, Ethier C, Oby ER, Hiatt SD, Wilder AM, Ko JH (2013). Intrafascicular stimulation of monkey arm nerves evokes coordinated grasp and sensory responses. J Neurophysiol.

[2] Javitt DC, Steinschneider M, Schroeder CE, Arezzo JC (1996). Role of cortical N-methyl-D-aspartate receptors in auditory sensory memory and mismatch negativity generation: implications for schizophrenia. Proc Natl Acad Sci.

[3] Pincze Z, Lakatos P, Rajkai C, Ulbert I, Karmos G (2001). Separation of mismatch negativity and the N1 wave in the auditory cortex of the cat: a topographic study. Clin Neurophysiol.

[4] Banoub M, Tetzlaff John E, Schubert A (2003). Pharmacologic and physiologic influences affecting sensory evoked potentials: implications for perioperative monitoring. Anesthesiology.

[5] Koelsch S, Heinke W, Sammler D, Olthoff D (2006). Auditory processing during deep propofol sedation and recovery from unconsciousness. Clin Neurophysiol.

[6] Richards CD (1983). Actions of general anaesthetics on synaptic transmission in the CNS. Br J Anaesth.

[7] Samra SK, Vanderzant CW, Domer PA, Sackellares JC (1987). Differential effects of isoflurane on human median nerve somatosensory evoked potentials. Anesthesiology.

[8] Peterson DO, Drummond JC, Todd MM (1986). Effects of halothane, enflurane, isoflurane, and nitrous oxide on somatosensory evoked potentials in humans. Anesthesiology.

[9] McPherson RW, Mahla M, Johnson R, Traystman RJ (1985). Effects of enflurane, isoflurane, and nitrous oxide on somatosensory evoked potentials during fentanyl anesthesia. Anesthesiology.

[10] Pathak KS, Ammadio M, Kalamchi A, Scoles PV, Shaffer JW, Mackay W (1987). Effects of halothane, enflurane, and isoflurane on somatosensory evoked potentials during nitrous oxide anesthesia. Anesthesiology.

[11] Sebel PS, Ingram DA, Flynn PJ, Rutherfoord CF, Rogers H (1986). Evoked potentials during isoflurane anaesthesia. Br J Anaesth.

[12] Hume AL, Durkin MA (1986). Central and spinal somatosensory conduction times during hypothermic cardiopulmonary bypass and some observations on the effects of fentanyl and isoflurane anesthesia. Electroencephalogr Clin Neurophysiol.

[13] Rehberg B, Rüschner R, Fischer M, Ebeling BJ, Hoeft A (1998). Concentration-dependent changes in the latency and amplitude of somatosensory-evoked potentials by desflurane, isoflurane and sevoflurane. Anasthesiol Intensivmed Notfallmed Schmerzther.

[14] Kaieda R, Maekawa T, Takeshita H, Maruyama Y, Shimizu H, Shimoji K (1981). Effects of diazepam on evoked electrospinogram and evoked electromyogram in man. Anesth Analg.

[15] Stone JL, Ghaly RF, Levy WJ, Kartha R, Krinsky L, Roccaforte P (1992). A comparative analysis of enflurane anesthesia on primate motor and somatosensory evoked potentials. Electroencephalogr Clin Neurophysiol.

[16] Scheepstra GL, de Lange JJ, Booij LH, Ros HH (1989). Median nerve evoked potentials during propofol anaesthesia. Br J Anaesth.

[17] Kuusela E, Raekallio M, Anttila M, Falck I, Mölsä S, Vainio O (2000). Clinical effects and pharmacokinetics of medetomidine and its enantiomers in dogs. J Vet Pharmacol Ther.

[18] Li BH, Lohmann JS, Schuler HG, Cronin AJ (2003). Preservation of the cortical somatosensory-evoked potential during dexmedetomidine infusion in rats. Anesth Analg.

[19] Lind NM, Moustgaard A, Jelsing J, Vajta G, Cumming P, Hansen AK (2007). The use of pigs in neuroscience: modeling brain disorders. Neurosci Biobehav Rev.

[20] Sauleau P, Lapouble E, Val-Laillet D, Malbert CH (2009). The pig model in brain imaging and neurosurgery. Animal.

[21] Andersen F, Watanabe H, Bjarkam C, Danielsen EH, Cumming P (2005). Pig brain stereotaxic standard space: mapping of cerebral blood flow normative values and effect of MPTP-lesioning. Brain Res Bull.

[22] Palmieri G, Farina V, Panu R, Asole A, Sanna L, De Riu PL (1986). Course and termination of the pyramidal tract in the pig. Arch Anat Microsc Morphol Exp.

[23] Nakamura M, Nishida S, Shibasaki H (1991). Deterioration of average evoked potential waveform due to asynchronous averaging and its compensation. IEEE Trans Biomed Eng.

[24] Arnfred SM, Lind NM, Gjedde A, Hansen AK (2004). Scalp recordings of mid-latency AEP and auditory gating in the Göttingen minipig: a new animal model in information processing research. Int J Psychophysiol.

[25] Benavides FD, Santamaria AJ, Bodoukhin N, Guada LG, Solano JP, Guest JD (2017). Characterization of motor and somatosensory evoked potentials in the yucatan micropig using transcranial and epidural stimulation. J Neurotrauma.

[26] Frasch MG, Walter B, Herry CL, Bauer R (2021). Multimodal pathophysiological dataset of gradual cerebral ischemia in a cohort of juvenile pigs. Sci Data.

[27] Maier S, Goebel U, Krause S, Benk C, Schick MA, Buerkle H (2018). Somatosensory and transcranial motor evoked potential monitoring in a porcine model for experimental procedures. PLoS ONE.

[28] Gurney M, Cripps P, Mosing M (2009). Subcutaneous pre-anaesthetic medication with acepromazine-buprenorphine is effective as and less painful than the intramuscular route. J Small Anim Pract.

[29] Grint NJ, Burford J, Dugdale AH (2009). Does pethidine affect the cardiovascular and sedative effects of dexmedetomidine in dogs?. J Small Anim Pract.

[30] Girard NM, Leece EA, Cardwell J, Adams VJ, Brearley JC (2010). The sedative effects of low-dose medetomidine and butorphanol alone and in combination intravenously in dogs. Vet Anaesth Analg.

[31] Craner SL, Ray RH (1991). Somatosensory cortex of the neonatal pig: I. Topographic organization of the primary somatosensory cortex (SI). J Comp Neurol.

[32] Craner SL, Ray RH (1991). Somatosensory cortex of the neonatal pig: II. Topographic organization of the secondary somatosensory cortex (SII). J Comp Neurol.

